# Case Report: Kidney allograft recovery after conversion to belatacept-based immunosuppression in simultaneous heart–kidney transplantation

**DOI:** 10.3389/fimmu.2026.1907833

**Published:** 2026-09-17

**Authors:** Elisa Gessaroli, Mario Sabatino, Angelodaniele Napoletano, Vasilika Samara, Daniele Vetrano, Chiara Baldovini, Valeria Grandinetti, Gaetano La Manna, Giorgia Comai, Luciano Potena, Valeria Corradetti

**Affiliations:** 1 Department of Medical and Surgical Sciences (DIMEC), Alma Mater Studiorum-University of Bologna, Bologna, Italy; 2 Heart Failure and Transplant Unit, IRCCS Azienda Ospedaliero-Universitaria di Bologna, Bologna, Italy; 3 Nephrology, Dialysis and Kidney Transplant Unit, IRCCS Azienda Ospedaliero-Universitaria di Bologna, Bologna, Italy; 4 Pathology Unit, IRCCS Azienda Ospedaliero-Universitaria di Bologna, Bologna, Italy

**Keywords:** alloimmunity, co-stimulation blockade, delayed graft function, donor-specific antibodies, heart transplant, kidney transplant, rejection, simultaneous heart-kidney transplantation

## Abstract

Renal allograft dysfunction after simultaneous heart-kidney transplantation (SHKT) is often multifactorial, reflecting perioperative hemodynamic instability, ischemia-reperfusion injury, alloimmune activation, and calcineurin inhibitor exposure. The therapeutic role of belatacept in this dual-organ setting remains poorly defined. We report the case of a 47-year-old woman with systemic lupus erythematosus, antiphospholipid syndrome, previous kidney allograft loss and heart failure, who underwent SHKT. The post-transplant course was complicated by persistent anuria requiring prolonged dialysis dependence, a high-titer *de novo* class II donor-specific antibody (DSA), and biopsy-proven cardiac antibody-mediated and cellular rejection. Despite intensive anti-rejection treatment, cardiac histology improved and DSA levels decreased, but renal allograft function did not recover. Belatacept was introduced at post-transplant week 12 with tacrolimus minimization. Thereafter, diuresis progressively resumed and dialysis was discontinued, with progressive improvement in renal function, while DSA became progressively undetectable. At 6 months, both grafts are functioning, serum creatinine is 1.4 mg/dL and no DSA rebound occurred. This case highlights the potential role of belatacept-based immunosuppression in the complex setting of SHKT with prolonged kidney allograft dysfunction.

## Introduction

Simultaneous heart-kidney transplantation (SHKT) has become an increasingly recognized option for selected patients with advanced heart failure and concomitant kidney disease, particularly when renal dysfunction is considered unlikely to recover after isolated heart transplantation (1). However, SHKT remains a highly complex surgical and immunological setting, in which effective alloimmune control must be balanced against drug-related nephrotoxicity. In this context, delayed graft function (DGF) of the kidney allograft has been reported in approximately one third of recipients, and persistent dialysis dependence may reflect overlapping hemodynamic, ischemic, immunological, and drug-related mechanisms rather than a single cause (2, 3). Immunosuppression strategies for SHKT are generally heart-transplant driven; induction commonly relies on rabbit antithymocyte globulin or interleukin-2 receptor antagonists, whereas maintenance therapy usually combines tacrolimus, mycophenolate mofetil (MMF), and corticosteroids. Previous heart transplant studies suggest that reducing or withdrawing calcineurin inhibitors (CNI) exposure may preserve renal function in selected patients, although these strategies may also increase rejection risk and require careful patient selection and monitoring (4, 5). When rejection occurs, treatment is usually guided by phenotype and targeted organ, including steroid pulses for cellular rejection and antibody-directed therapies for antibody-mediated rejection (ABMR) (6). After SHKT, rejection rates are generally reported to be lower than after isolated heart or kidney transplantation. However, SHKT does not confer the predictable immunological shielding observed in heart-liver transplantation, and *de novo* donor-specific antibodies (DSA) and ABMR may occur, particularly in HLA-sensitized recipients (1). Maintaining a balance between adequate immunosuppressive regimens and multiple risk factors for acute and chronic kidney injury is increasingly relevant as transplant candidates are older and more medically complex, often with prior transplantation, prolonged dialysis exposure, and multiple comorbidities. In this setting, belatacept, a selective costimulation blocker targeting the CD28–CD80/86 pathway, is clinically relevant because it offers a CNI-sparing strategy while maintaining T-cell costimulation blockade. Its use has been associated with improved renal outcomes in kidney transplantation and is emerging as a CNI-sparing strategy in heart transplantation as well (7–9). However, its role in multi-organ transplantation remains poorly defined, and evidence in combined heart-kidney transplant is very limited (10). We report a case of prolonged kidney allograft dysfunction after SHKT in which belatacept was introduced with concomitant CNI minimization. Following this intervention, urine output and kidney allograft function progressively recovered, while DSA continued to decline, becoming undetectable. This case highlights the potential role of belatacept-based immunosuppression in SHKT, where the risk of kidney allograft dysfunction requires careful balance between renal preservation and alloimmune control.

## Case summary

We report the case of a 47-year-old woman with systemic lupus erythematosus, antiphospholipid syndrome, and prior kidney transplantation on chronic hemodialysis treatment who underwent a SHKT at the Bologna University Hospital (Italy). Her cardiac disease was characterized by advanced valvular pathology requiring multiple surgical interventions and subsequent heart failure. The donor was a 60-year-old Maastricht III donation-after-circulatory-death (DCD) donor who died from cerebral hemorrhage (KDPI 70%; serum creatinine 0.53 mg/dL). Despite a prior transplant, the recipient calculated panel reactive antibody (cPRA) was 0%. The combined transplant was performed in the presence of a negative T-lymphocyte crossmatch and a weakly positive B-lymphocyte crossmatch, in the absence of detectable DSA. Induction immunosuppression consisted of a single 75-mg dose of thymoglobulin, with further doses withheld because of severe thrombocytopenia, while maintenance immunosuppression included tacrolimus (target level 10–15 ng/mL), MMF, and corticosteroids. The early post-transplant course was characterized by DGF of the kidney allograft, with prolonged dialysis dependence and persistent anuria. Moreover, the patient developed *de novo* high-titer class II DSA (anti-DQ5, peak mean fluorescence intensity [MFI] 22,400) by post-operative week 2 and biopsy-proven cardiac rejection, including both antibody-mediated rejection and acute cellular rejection (ACR 2R plus pAMR 1) (**Figure 1**). Kidney allograft biopsy was deferred because of the low platelet count, which reached a nadir of 31,000/mm³ on post-operative day (POD) 7. The thrombocytopenia was considered multifactorial, involving immunosuppressive drug exposure, perioperative platelet consumption related to major cardiac surgery and subsequent development of bacteremia. Thrombotic microangiopathy was also considered because of transient laboratory evidence of hemolysis, with LDH reaching a peak of 589 U/L and haptoglobin <30 mg/dL; however, the hemolytic abnormalities rapidly resolved by week 1. Other potential causes, including thrombotic thrombocytopenic purpura, heparin-induced thrombocytopenia, and primary immune-mediated thrombocytopenia, were excluded. Treatment included high-dose corticosteroids, plasmapheresis, intravenous immunoglobulins, and complement inhibition with eculizumab (**Figure 2**). Rituximab was withheld due to concurrent K. pneumoniae bacteremia treated with antibiotics and mild cytopenias. Although these interventions resulted in a reduction of DSA levels - which nevertheless remained positive (MFI 4,400) - and improvement in cardiac histology, kidney graft function did not recover. Indeed, seventy-five days after transplantation the patient was still anuric and dialysis dependent. Following improvement in platelet count, a kidney graft biopsy was performed at post-transplant week 12, showing no evidence of active cellular or antibody-mediated rejection according to Banff criteria, but rather diffuse ischemic acute tubular injury associated with moderate chronic tubulointerstitial damage and severe vascular fibrous intimal thickening. A repeat endomyocardial biopsy performed the following day revealed grade 2 acute cellular rejection without features of antibody-mediated rejection. To minimize CNI toxicity and limit progression of chronic injury, a belatacept-based regimen was therefore initiated, with a dosing schedule of 10 mg/kg on days 0, 14, and 28, followed by 6 mg/kg monthly. Tacrolimus exposure was concurrently reduced to target trough levels of 6–7 ng/mL, while steroids and MMF were maintained. Following belatacept initiation, DSA levels continued to decline and eventually became undetectable (**Figure 2**). In parallel, after a prolonged period of anuria, the patient experienced gradual recovery of urine output, allowing discontinuation of dialysis and subsequent stabilization of renal function. A second kidney biopsy at week 16 revealed mild T cell–mediated rejection, successfully treated with steroid pulses. At 6 months after transplantation, both grafts are functioning and serum creatinine is 1.4 mg/dL. Notably, the latest endomyocardial biopsy showed no evidence of antibody-mediated or cellular rejection (**Figure 1**) and DSA are still undetectable.

**Figure 1:**
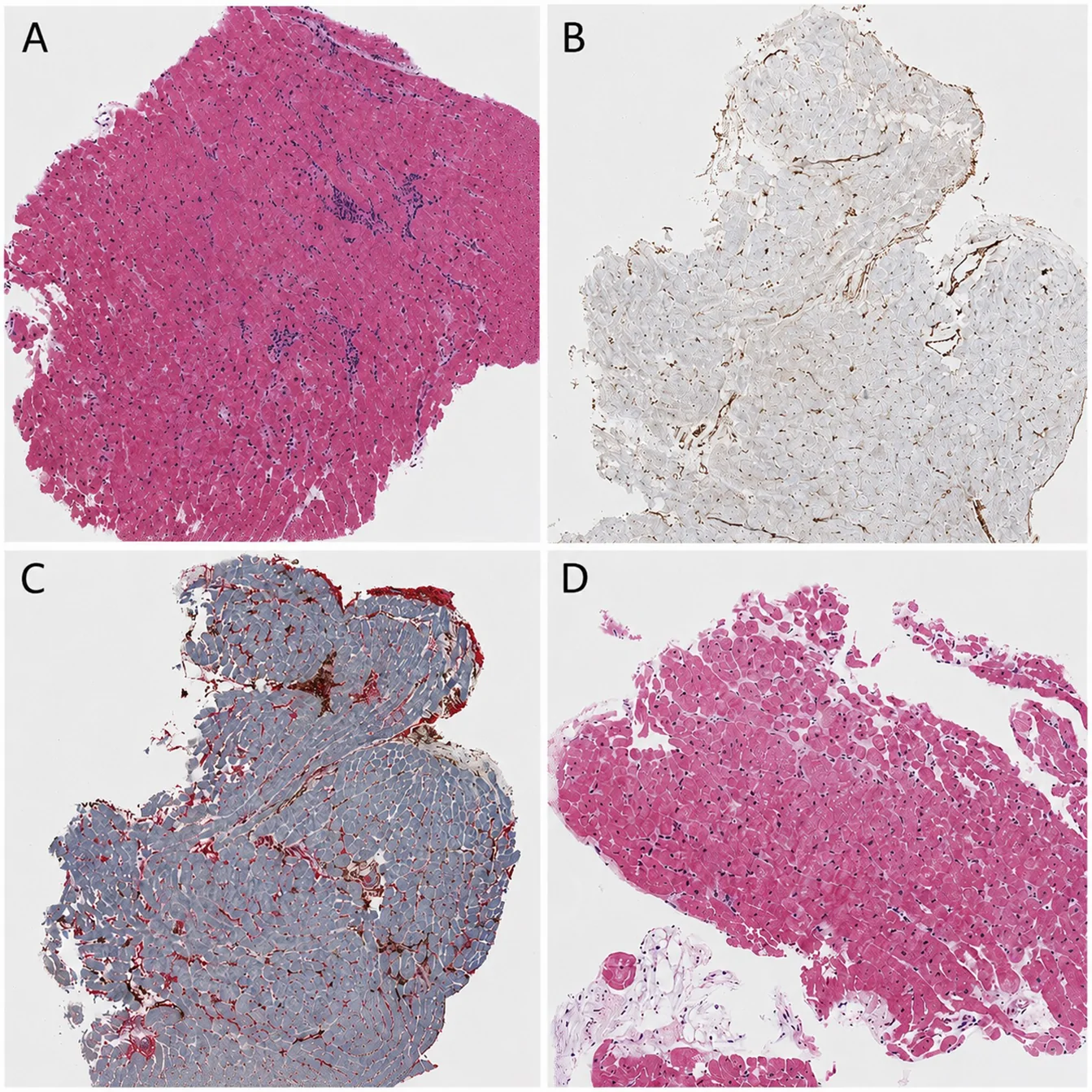
Representative endomyocardial biopsy findings. **(A–C)**. Diagnostic endomyocardial biopsy showing moderate acute cellular rejection (ACR grade 2R) with focal interstitial lymphocytic infiltrates and associated myocyte injury [**(A)**, H&E], strong multifocal-to-diffuse C4d capillary staining involving >50% of the microvasculature [**(B)**, C4d immunohistochemistry], and increased CD68-positive macrophages in the myocardial interstitium with focal intravascular macrophage accumulation within capillaries and small venules [**(C)**, CD68/CD34 duplex immunohistochemistry]. **(D)** Follow-up endomyocardial biopsy showing no interstitial lymphocytic infiltrates, consistent with absence of acute cellular rejection (ACR grade 0R; H&E).

**Figure 2:**
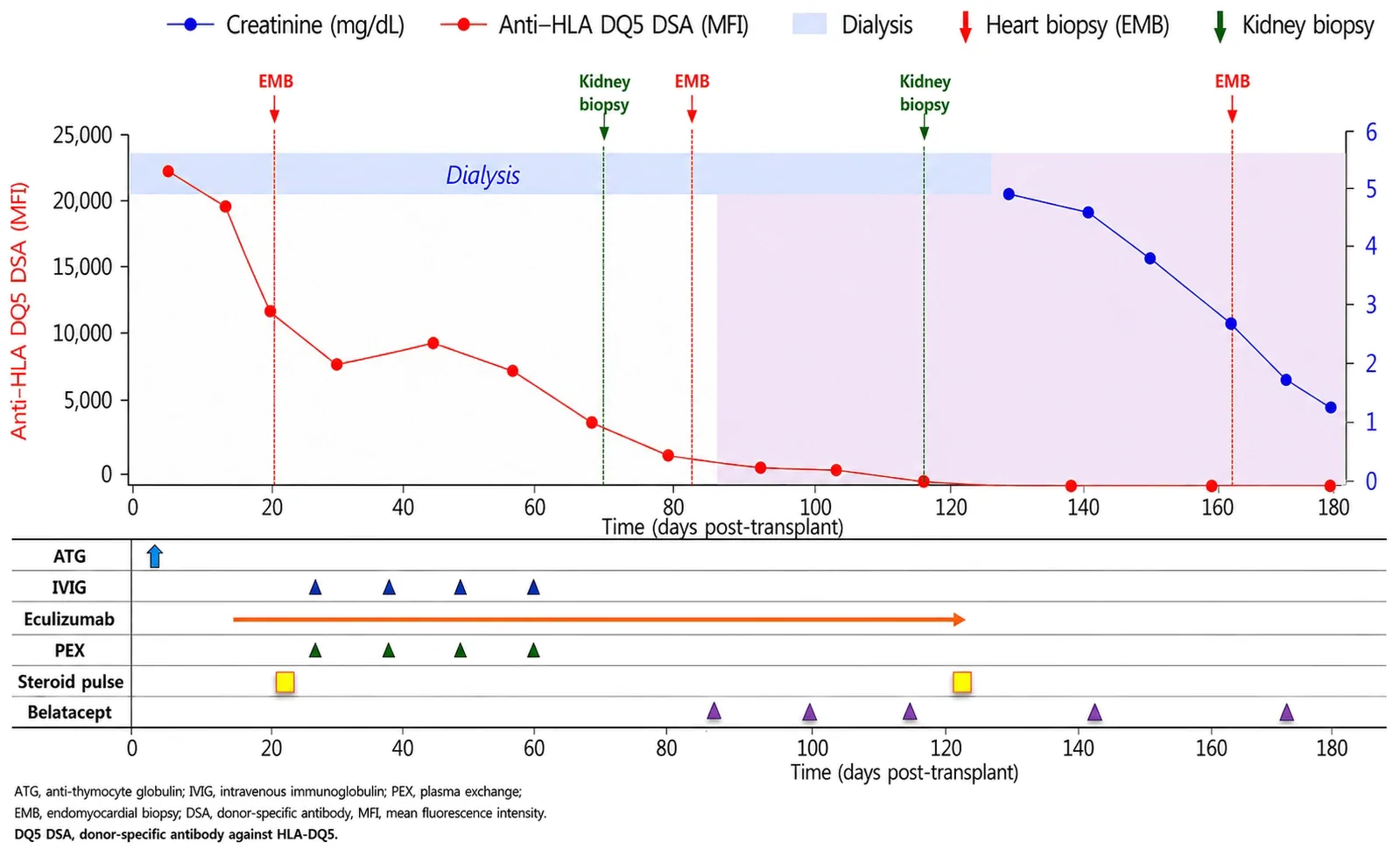
Clinical course after simultaneous heart–kidney transplantation (SHKT). Temporal evolution of anti-HLA DQ5 DSA levels, kidney allograft function, rejection episodes, biopsies, and immunosuppressive treatments after SHKT. The early post-transplant course was complicated by prolonged dialysis-dependent DGF, high-titer *de novo* DSA, and biopsy-proven cardiac rejection. Following belatacept initiation and tacrolimus reduction, DSA levels continued to decline and eventually became undetectable, while kidney allograft function gradually recovered allowing dialysis discontinuation.

## Discussion

SHKT is a challenging setting in solid organ transplantation, as it requires a careful balance between effective alloimmune control and preservation of renal graft function, in a context characterized by major surgical, hemodynamic, and immunological complexity. Such complexity becomes greater when recipients present several comorbidities and a long disease history. In this context, DGF is unlikely to reflect a single mechanism, but rather the convergence of ischemia-reperfusion injury, perioperative instability, alloimmune activation, and CNI-related nephrotoxicity. Although belatacept is now an established CNI-sparing strategy in kidney transplantation, its role in heart and multi-organ transplantation remains largely unexplored. Against this background, the presented case is clinically relevant because it illustrates the potential value of a belatacept-based approach in an exceptionally complex SHKT recipient with prolonged kidney graft dysfunction and severe early alloimmune complications. SHKT is increasingly recognized as a valuable option for selected patients with advanced cardiac dysfunction and concomitant kidney disease, although candidate selection remains challenging (1). Overall, reported outcomes are acceptable, with pooled survival rates of 95% at 30 days, 81% at 1 year, and 71% at 5 years, while registry data suggest that SHKT may confer a survival advantage over isolated heart transplantation in dialysis-dependent recipients and in selected non–dialysis-dependent candidates with severely reduced kidney function (2). Yet, kidney injury remains a major determinant of prognosis even after isolated heart transplantation, where acute kidney injury occurs in 40%–70% of recipients and early dialysis is required in a substantial proportion of recipients (10-12%), and both are associated with worse short- and long-term outcomes (11). In the specific setting of SHKT, delayed kidney graft function is relatively common, affecting approximately one third of recipients, whereas pooled rejection rates are not particularly high (17% for cardiac rejection and 13% for renal rejection) (3). However, our patient developed early *de novo* high-titer class II DSA and biopsy-proven cardiac rejection in the context of persistent kidney allograft dysfunction and anuria. Unfortunately, initial kidney biopsy had to be deferred because of severe thrombocytopenia. The patient promptly received intensive treatment for both cellular and antibody-mediated cardiac rejection (including anti-C5 therapy in the presence of C4d positivity at heart biopsy) which would also have been expected to treat a possible concomitant renal alloimmune process. Yet, despite improvement in cardiac histology and a reduction in DSA MFI, no meaningful renal recovery was observed, and the patient remained dialysis dependent for months. When kidney biopsy was eventually feasible, it did not show active rejection, but rather evidence of potential calcineurin inhibitor-related nephrotoxicity, further supporting the multifactorial nature of graft dysfunction in this case. Usually, tacrolimus trough targets after heart transplantation are intentionally maintained at relatively high levels in the early post-transplant phase, with ISHLT recommending 10–15 ng/mL during the first 60 days (12). Thus, a potential contribution of tacrolimus to graft dysfunction is biologically plausible, as higher whole-blood tacrolimus concentrations have been associated with early acute kidney injury after heart transplantation. In liver transplantation, lower early tacrolimus exposure has been associated with less renal impairment, with no statistically significant difference in rejection rates (13, 14). These considerations have prompted interest in CNI-sparing strategies. In heart transplantation, mechanistic target of rapamycin (mTOR) inhibitor-based regimens have been used as alternative approaches, although renal outcomes have varied according to the timing and extent of CNI reduction or withdrawal. In the randomized SCHEDULE trial, early cyclosporine withdrawal under everolimus resulted in improved measured glomerular filtration rate (GFR) compared with continued cyclosporine, but was associated with a higher incidence of acute cellular rejection. In contrast, the CECARI trial, which evaluated everolimus with CNI withdrawal versus continued CNI, did not demonstrate a significant renal benefit in the primary intention-to-treat analysis, although an on-treatment analysis showed improved renal function. The composite safety endpoint, including treated acute rejection, was not significantly different between groups (4, 15). Consistent with these findings, a meta-analysis of trials in heart transplantation reported improved renal function with CNI-sparing mTOR-based regimens, but a higher incidence of acute cellular rejection compared with CNI-based strategies was observed (16). These data suggest that the renal benefit of mTOR-based CNI-sparing strategies may depend on the clinical context and must be balanced against the risk of rejection associated with more intensive CNI reduction or withdrawal. Data in combined heart–kidney transplantation (HKT) remain limited. In a retrospective cohort of 302 heart transplant recipients, including 40 HKT, conversion from CNI-based to sirolimus-based immunosuppression was not associated with significant changes in estimated GFR or proteinuria (17). Costimulation blockade therefore represents another potential therapeutic option in this setting, but evidence in heart transplantation remains scarce and is currently limited to observational studies and retrospective single-center cohorts (8–10). In these studies, belatacept has primarily been introduced in patients with renal dysfunction or concerns regarding CNI toxicity, with tacrolimus either minimized or withdrawn and renal function generally stable or improved during follow-up. No randomized comparative data are available, and the superiority of belatacept over mTOR inhibitors - associated with CNI minimization or withdrawal - has not been established in either heart transplantation or HKT. Moreover, experience with the use of belatacept in combined transplantation remains particularly limited, consisting mainly of small observational series and isolated reports in other multiorgan settings (18, 19). A comparative cohort of belatacept-treated heart transplant recipients included only 2 HKT recipients, and outcomes for this subgroup were not reported separately (10). Beyond its CNI-sparing effect, belatacept may influence humoral alloimmunity. Experimental and translational studies suggest that belatacept, by blocking CD28-mediated costimulation through CD80/86, may modulate T follicular helper–B-cell interactions and germinal-center responses, thereby providing a potential mechanistic basis for effects on DSA production (20–22). In *post hoc* analyses of the phase III BENEFIT and BENEFIT-EXT kidney transplantation trials, belatacept-based immunosuppression was associated with a lower incidence of *de novo* DSA than cyclosporine-based immunosuppression. Among recipients who developed *de novo* DSA, antibody strength, assessed by MFI, was generally lower with belatacept (23). However, these findings were obtained in kidney transplant populations and cannot be directly extrapolated to SHKT recipients. In a recent observational heart transplant and HKT cohort, none of 12 belatacept-treated patients developed *de novo* DSA, compared with 19% of 48 tacrolimus-treated controls, with no significant difference in rejection (10). Our patient had no detectable preformed DSA, but developed a high-titer *de novo* anti-DQ5 DSA early after transplantation. The initial reduction in DSA occurred before belatacept initiation and followed treatment for antibody-mediated rejection. Notably, DSA levels continued to decline after belatacept initiation and eventually became undetectable. Although this temporal sequence is compatible with a possible contribution of costimulation blockade to subsequent control of the humoral alloimmune response, it does not establish a specific effect of belatacept. DSA reduction had already begun after plasmapheresis, intravenous immunoglobulins, and eculizumab, while tacrolimus was continued at reduced exposure during belatacept therapy. To our knowledge, this appears to be the first detailed case report describing the use of belatacept with CNI minimization for prolonged kidney graft dysfunction after simultaneous heart-kidney transplantation complicated by organ rejection. In our case, belatacept introduction together with tacrolimus minimization was followed by progressive recovery of urine output, discontinuation of dialysis, and subsequent restoration of kidney graft function. However, the temporal association does not establish a causal relationship, and the contribution of reduced CNI exposure and spontaneous recovery from ischemic tubular injury cannot be excluded. We chose tacrolimus minimization rather than complete withdrawal because of the previous cardiac allograft rejection and the need to maintain adequate immunological protection. Indeed, a mild T cell–mediated rejection was observed, successfully managed with steroid pulses. Overall, our case supports the biological plausibility of CNI-sparing strategies to preserve renal function, and emerging observational data suggest that belatacept may represent a promising option in selected recipients. Remarkably, diuresis resumed nearly 3 months after transplant, a timeframe at which such prolonged dysfunction is often considered highly suggestive of primary nonfunction. However, in SHKT the evidence base remains exceptionally limited, and any inference regarding efficacy, optimal timing, and degree of CNI minimization or withdrawal is still largely speculative. Future prospective multicenter studies are needed to specifically address the role of belatacept in this highly complex dual-organ setting.

## Data Availability

The original contributions presented in the study are included in the article/supplementary material. Further inquiries can be directed to the corresponding author.
